# Lower limb biomechanical differences between jumps with different number of rotations in youth figure skaters

**DOI:** 10.3389/fbioe.2025.1606817

**Published:** 2025-09-01

**Authors:** Ami Koga, Xiaotian Bai, Yuanyuan Jia, Jingmin Liu

**Affiliations:** ^1^ Division of Sports Science and Physical Education, Tsinghua University, Beijing, China; ^2^ School of Sport Science, Beijing Sport University, Beijing, China

**Keywords:** figure skating, biomechanics, lower limbs, youth, jumps

## Abstract

**Objective:**

The purpose of this study was to examine the differences in the lower-limb muscle activities and kinematics between figure skating Axel type jumps with different number of rotations in youth figure skaters. We hypothesized that skaters would exhibit increased lower limb flexion during jump propulsion phase, lower limb extension at take-off and greater muscle activation levels as jump rotation increases.

**Methods:**

Eleven youth figure skaters (age: 12 ± 4.29 years; height: 146.82 ± 17.71 cm; body mass: 37.02 ± 14.47 kg) performed Waltz Jump (0.5 rotations), Single Axel Jump (1.5 rotations), and three of them additionally performed Double Axel Jump (2.5 rotations). Lower-limb kinematics were recorded using two high-speed cameras. Muscle activities of Rectus Femoris, Long Head of Biceps Femoris, Tibialis Anterior, Lateral Gastrocnemius, and Medial Gastrocnemius of both legs were measured. The differences between the jumps were compared using paired samples t-test. Comparison of EMG data between different muscles parts was performed by One-way ANOVA. Due to limited data, Double Axel jump was compared with descriptive analysis.

**Results:**

More difficult Axel type jump had higher jump height, shorter jump distance, faster jump take-off vertical velocity, and greater hip flexion during propulsion phase. The RMS and iEMG values of the left medial and lateral gastrocnemius and right tibialis anterior increased as the jump difficulty increased. Moreover, there were significant differences between different muscle parts RMS values and iEMG values in both Waltz jump and Single Axel jump (p < 0.01). Biceps femoris and rectus femoris indicated to have the highest RMS values and iEMG values in Waltz jump and Single Axel jump.

**Conclusion:**

More difficult Axel type jumps require greater hip flexion during propulsion phase and greater activities in hamstrings, quadriceps and tibialis anterior before jump take-off. Youth figure skaters can improve jump height, take-off vertical velocity and overall qualities of jumps by enhancing multi-joint movement, muscle coordination and take-off leg strength. These findings provide insights into the lower-limb biomechanical characteristics of figure skating jumps, and potentially leading to refinement of training programs for the youth figure skaters to optimize jump performances and to reduce potential lower extremity injuries.

## 1 Introduction

Figure skating is one of the popular sports in winter Olympic sport categories. According to International Skating Union (ISU), there are six types of jumps in figure skating: Toe-loop, Loop, Salchow, Flip, Lutz, Axel. Jumps are categorized according to how the skater performs jump take-off and lands on the ice. In addition, the difficulty of a jump is based on both the type of jump and the total number of rotations in the air.

Axel Jump is the most difficult jump with an extra half turn in the flight time compared to the other jumps due to forward jump take-off, and backwards landing, where the jumping leg and the landing leg must be opposite legs. Axel type jump is the first jump taught in figure skating, and it is the most critical jump considering its technical aspects and jump technical scores (International Skating Union, 2024). Waltz jump is a jump that leads to the Axel jump, the first jump skaters learn, and it is a half rotation jump (Hines, 2006). Half rotation jump is called Waltz, one and half rotations jump is called Single Axel, two and half rotations jump is called Double Axel, and three and half rotations jump is called Triple Axel.

Figure skaters need to be equipped with good physical qualities, as well as artistic and musical senses. These qualities need to be developed over a long period of time; thus, a large amount of training and early specialization is necessary. (Baker, 2003; Cattle et al., 2023). Youth is not only the golden period for developing fundamental skills, but also the most sensitive phase to body development and sports injuries. Lower limb overuse injuries are common among junior elite skaters with poor movement technique and high impact from repetitive jumps (Dubravcic-Simunjak et al., 2003).

Biomechanical research has been widely conducted in the sport industry. Biomechanical analysis procedures include kinematic, electromyography (EMG) and kinetic analysis are the most used research method for sport performance optimization, injury reduction and equipment development (Taborri et al., 2020; Kos and Umek, 2019; Bruening et al., 2018). Such analysis is also relevant and very critical in competitive sport such as figure skating (Lockwood et al., 2006). In the previous studies of Axel jump, while no differences in jump height and vertical velocity at jump take-off were found between different Axel jumps, and jumps with more rotations are claimed to be completed by the increase in jump rotation speed (King, 2005; King et al., 1994), other studies found that there were increases in the jump height, jump rotational speed, jump flight time, angular velocity and decrease in the jump distance as the jump difficulty increases (Hisako et al., 2004). Moreover, increasing the maximum jump height was found to be the key to succeed Quadruple Axel jump, Triple Axel jump is done by more efficient angular momentum (Hirosawa, 2004). Muscle activity levels of medial gastrocnemius, rectus femoris, biceps femoris, and adductor muscle were found to have greater activities in jumps with more rotations, whereas a similar activation pattern was observed for the adductors in all jumps (Taylor and Psycharakis, 2009). The gastrocnemius lateralis, rectus femoris, biceps femoris and vastus lateralis muscles presented greater activity and longer duration during roller skating Double Axel jump than Single Axel jump figure skating jumps (Pantoja et al., 2014). Unique activation patterns and muscle activity levels in jumps with different rotations suggests that figure skaters need to have the ability to change the technique of the jumps according to the desired jump difficulty with different number of rotations.

Overall, there are mixed findings in the kinematics of Axel jump of different number of rotations, and strategies employed by youth skaters to acquire new jumps are not well discovered yet. Despite figure skating’s technical difficulties and high risks of lower-limb injuries (Rauer et al., 2022; Madsen et al., 2024; Jederström et al., 2021), most studies have focused on kinematical aspect of Axel type jumps, and very few studies combined both kinematics and EMG analysis to explore characteristics of jumps. Therefore, this study aims to explore the kinematical characteristics, muscular activation levels and patterns of figure skating Axel type jumps with different number of rotations in youth figure skaters. The findings could provide deeper understanding on the similarities and differences between jumps with different rotations to aid in developing effective training programs and injury prevention strategies targeting youth figure skaters to optimize performance and prevent potential lower limb injuries.

We hypothesized that as jump rotation increases, skaters would exhibit increased lower limb flexion during propulsion phase, lower limb extension at take-off and greater muscle activation levels.

## 2 Methods

### 2.1 Participants

Eight female and three male skaters, total of eleven youth figure skaters (age: 12 ± 4.29 years; height: 146.82 ± 17.71 cm; body mass: 37.02 ± 14.47 kg) performed Waltz Jump (0.5 rotations), Single Axel Jump (1.5 rotations), and three of them additionally performed Double Axel Jump (2.5 rotations). Given the limited number of youth skaters capable of performing this advanced jump safely, we chose to include these data to provide initial insights into its unique biomechanical demands. All skaters were competing at reginal or national levels, with an average training experience of 5 years, and regular skating practice for at least 10 h per week. All skaters were right-handed. Jump were performed with the left foot as the jump take-off leg, the right foot as the landing leg, and the direction of jump rotation was counterclockwise. Skaters were free of injury and illness during the study. All participants and their parents provided written informed consent prior to the study.

### 2.2 Experimental protocol

**FIGURE 1:**
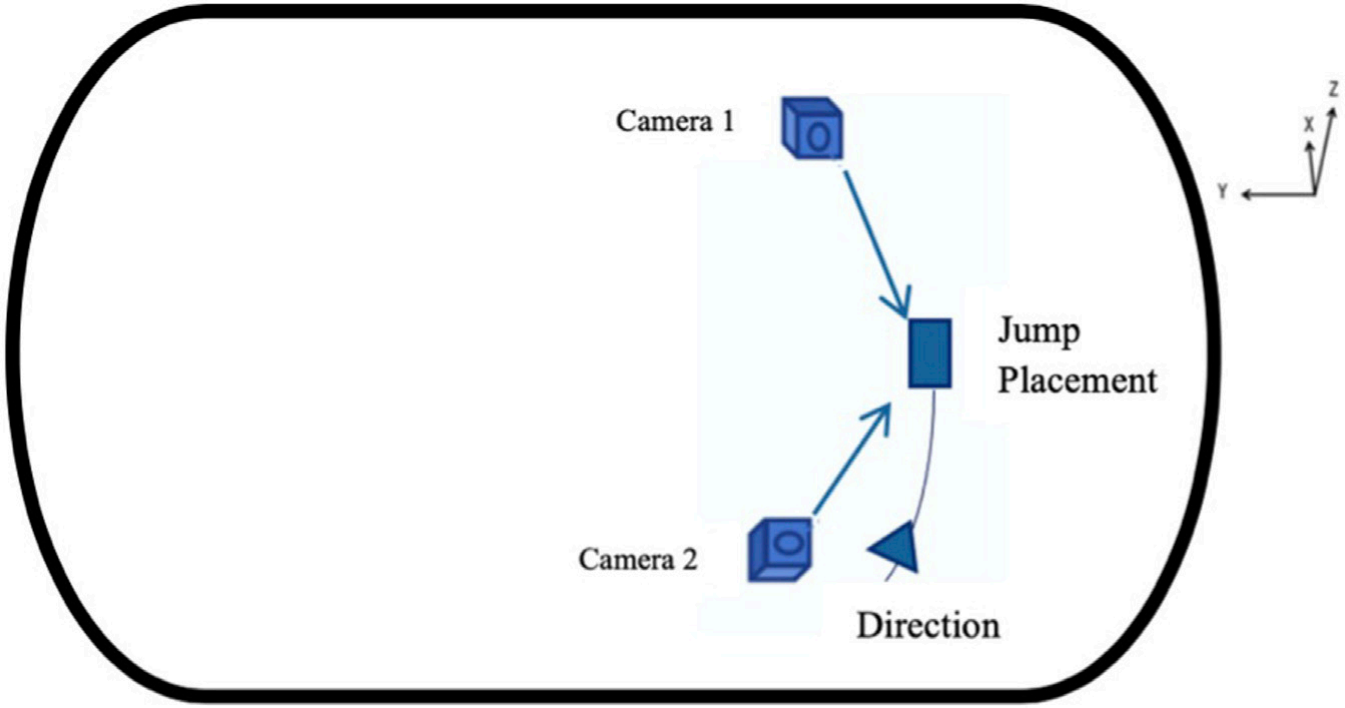
Jump kinematic data acquisition site layout on ice arena.

Two high-speed cameras (Sony NEX- FS700, at frame rate of 100 Hz) were set up in the ice rink according to the athlete jump placement to record skaters’ jumps kinematics. The cameras were positioned at angle of 110°. The camera placements can be seen in Figure 1. The same standardized calibration procedure was conducted before and after each final using a rigid three-dimension calibration frame with 28 reference points was used. Reflective markers were used to mark individual lower limb joints for modeling purposes. Two cameras and Direct Linear Transformation (DLT) method was used for 3D coordinates reconstruction (Cohen et al., 2017; Pueo, 2016). The X-, Y-, and Z-axes of the 3D coordinate system were the sagittal, coronal, and vertical axis directions, respectively. Ten surface electromyography (EMG) sensors (Delsys Trigno Wireless EMG System, at sampling frequency of 2000 Hz) were placed on the skin overlying the muscle belly of the lower extremity muscles including Rectus Femoris (RF), Long Head of Biceps Femoris (BF), Tibialis Anterior (TA), Lateral Gastrocnemius (LG), and Medial Gastrocnemius (MG) of both legs to assess neuromuscular patterns and muscle activity level at jump’s take-off and landing. Sensor placements followed the SENIAM guidelines (Hermens et al., 2000), and all placements were performed by the same person to ensure consistency. To minimize motion artifacts, we used double-sided tapes, and participants’ skins were shaved and cleaned with alcohol wipes before sensor placement.

Prior to the test, the athlete performed a few trial jumps to check all the sensors and markers were fixed and does not interfere with the athlete’s ability to complete the movements. After a warm-up, skaters performed each jump three times for actual data collection session.

### 2.3 Data processing

Manual digitizing was conducted in SIMI Motion (version 9.2.2, Simi Reality Motion Systems GmbH, Germany) to obtain kinematic data form jump propulsion phase, take-off phase, flight phase and landing phase. To minimize the error, all the videos were manually digitizing by one person. The main kinematic parameters obtained include the time parameters of each phase of the jump, the angles of the hip, knee and ankle, the jumping height, the distance, and the spatial parameters such as the peak vertical velocity of the jump. For this study, the onset of the movement was defined as the instant of the left foot placed on the ice at the beginning of the jump during propulsion phase. The end of movement was defined as when athletes stabilized after jump landing. The overall jump time ranged average of 2.5 s. EMG data collection, the initial screening and export of the data were conducted using EMGWorks Acquisition software version 4.8.0, developed by Delsys. Raw EMG signals were band-pass filtered at 20–500 Hz using a ButterWorth filter made in Python 3.11. 5 to remove artifacts before subsequent data processing, and the filtered data were normalized to the mean peak EMG value obtained in each movement (Maddox et al., 2022). After full-wave rectification, envelope extraction, root-mean-square amplitude (RMS) and integrated EMG (iEMG) were calculated. Each muscle was considered activated when its EMG amplitude reached more than 10% of its peak value in each movement.

### 2.4 Statistical analysis

Prior to statistical analysis, the normality of all variables was assessed using the Shapiro–Wilk test. Normally distributed kinematic data and EMG data of different jumps were compared using paired samples t-test. Non-normal data were analyzed statistically using the non-parametric Wilcoxon signed-rank test. Due to limited data, Double Axel jump was compared with descriptive analysis. Comparison of EMG data between different muscles was first performed by One-way ANOVA, and if the difference between the data was found to be significant, the Tukey HSD *post hoc* test was applied. All statistical analysis was conducted using Python 3.11.5. Significance level was set at p < 0.05.

## 3 Result

### 3.1 Kinematics

Firstly, all kinematical variables and terms used and analyzed can be seen in Table 1. As shown in Table 2, there was a significant difference between the take-off time of Waltz and Single Axel jump, with Waltz jump having a longer take-off time than Single Axel jump (p < 0.05). There was also a significant difference between flight time of Waltz jump and Single Axel Jump, with Waltz jump having a longer flight time than Single Axel jump (p < 0.01). There was no significant difference in the propulsion time and landing time. Propulsion time and take-off time of Double Axel jump are approximately the same as Waltz jump and slightly longer than Single Axel jump, the flight time and landing time were the longest in Double Axel jump. There was a significant difference between the jump height of Waltz jump and Single Axel jump (p < 0.01). Single Axel jumps with higher jump height. There was also a significant difference between the jump distance of Waltz jump and Single Axel jump (p < 0.01). Waltz jump with longer jump distance. There was also a significant difference between the jump take-off vertical velocity of Waltz jump and Single Axel jump, Single Axel jump with faster take-off vertical velocity (p < 0.01). Out of all jumps, Double Axel jump showed the highest jump height, shortest jump distance, and fastest jump take-off vertical velocity.

**TABLE 1:** Definition for all variables used and analyzed.

Variable	Definition
Propulsion Phase	The moment from jump take-off leg touches down ice to body reaching to the lowest point before jump, in another words, the end of knee flexion
Take-off phase	The moment from body reached to the lowest point before jump to moment of take-off leg leaving the ice. From the beginning of knee extension to the moment of toe-off
Flight phase	The moment while skaters are in air completing jump rotations. From the toe-off to initial ground contact on landing foot
Landing phase	From the first contact of the landing foot to the point where the body reaches stable posture, defined by minimal vertical displacement of the center of mass
Jump height	The change in height from jump take-off to maximum height skater reached during flight phase
Jump horizontal distance	The horizontal distance skater travelled during flight phase
Take-off vertical speed	The peak vertical velocity of the centre of mass at the instant of take-off leg leaving the ice
Hip Angle	The angle of the thigh relative to the torso
Knee Angle	The angle of the thigh relative to the shank
Ankle Angle	The angle of the shank relative to the foot
Propulsion Angle	Joint angle at the end of propulsion phase
Take-off Angle	Joint angle at the end of take-off phase
Landing Angle	Joint angle at the end of landing phase
Range of Motion (ROM)	Change in joint angle from the beginning of each phase to the end of the phase

**TABLE 2:** Variables (mean ± SD) representing the kinematics and spatiotemporal characteristics of Waltz, Single Axel and Double Axel jump.

Variable	Waltz (n = 11)	Single Axel (n = 11)	Double Axel (n = 3)	*p*-value
Temporal				
Propulsion time	0.45 ± 0.07	0.44 ± 0.06	0.45 ± 0.03	0.57
Take-off time	0.37 ± 0.05	0.34 ± 0.05	0.36 ± 0.08	0.012*
Flight time	0.36 ± 0.10	0.40 ± 0.07	0.51 ± 0.04	0.003*
Landing time	0.48 ± 0.16	0.57 ± 0.11	0.61 ± 0.09	0.05
Spatial				
Jump height	0.18 ± 0.09	0.24 ± 0.10	0.36 ± 0.13	0.001*
Jump distance	1.51 ± 0.54	1.14 ± 0.37	1.11 ± 0.07	0.004*
Take-off vertical velocity (m·s^–1^)	1.77 ± 0.46	2.24 ± 0.42	2.73 ± 0.31	<0.001*

*p*-value represents statistical comparisons between Waltz jump and Single Axel jump only.

* indicates *p* < 0.05.

As shown in Table 3, there was significant difference between the hip angle at propulsion phase of Waltz jump and Single Axel jump (p = 0.04). Double Axel jump showed the smallest propulsion phase hip and knee angle, and the largest hip ROM compared to the other two jumps descriptively. Therefore, as jump difficulty increased, propulsion phase hip and knee flexion increased, and hip ROM tend to increase.

**TABLE 3:** Variables (mean ± SD) representing the lower limb joint angle characteristics of Waltz, Single Axel and Double Axel jump.

Variable	Waltz (n = 11)	Single Axel (n = 11)	Double Axel (n = 3)	*p*-value
Propulsion angle				
Hip Angle	135.86 ± 20.63	125.62 ± 15.70	92.07 ± 4.38	0.040*
Knee Angle	129.23 ± 15.37	125.99 ± 8.22	114.33 ± 4.38	0.260
Ankle Angle	73.65 ± 19.29	67.69 ± 11.57	70.17 ± 20.37	0.485
Hip ROM	12.12 ± 7.48	12.61 ± 11.25	19.37 ± 8.47	0.881
Knee ROM	17.09 ± 8.55	18.25 ± 13.61	17.77 ± 10.79	0.756
Ankle ROM	18.37 ± 14.33	21.03 ± 12.99	11.97 ± 14.60	0.673
Take-off Angle				
Hip Angle	164.59 ± 8.05	156.46 ± 16.53	164.17 ± 8.89	0.419
Knee Angle	161.72 ± 14.65	156.68 ± 16.56	162.93 ± 8.89	0.100
Ankle Angle	91.02 ± 16.01	83.89 ± 11.01	81.43 ± 9.38	0.321
Hip ROM	28.73 ± 18.80	30.85 ± 24.50	72.1 ± 10.75	0.797
Knee ROM	32.49 ± 16.19	30.69 ± 12.81	48.6 ± 12.07	0.800
Ankle ROM	17.37 ± 23.74	16.20 ± 14.10	11.27 ± 21.34	0.910
Landing Angle				
Hip Angle	107.6 ± 24.07	97.15 ± 14.66	105.07 ± 21.52	0.330
Knee Angle	124.18 ± 18.60	114.66 ± 12.47	126.27 ± 21.52	0.258
Ankle Angle	62.20 ± 12.01	64.12 ± 13.89	66.80 ± 3.87	0.716
Hip ROM	53.65 ± 30.27	63.06 ± 22.14	61.53 ± 16.76	0.424
Knee ROM	31.36 ± 18.49	43.05 ± 19.09	27.13 ± 11.13	0.170
Ankle ROM	25.07 ± 23.85	15.89 ± 18.59	11.07 ± 8.21	0.388

*p*-value represents statistical comparisons between Waltz jump and Single Axel jump only.

* indicates *p* < 0.05.

### 3.2 Muscle activation patterns

**FIGURE 2:**
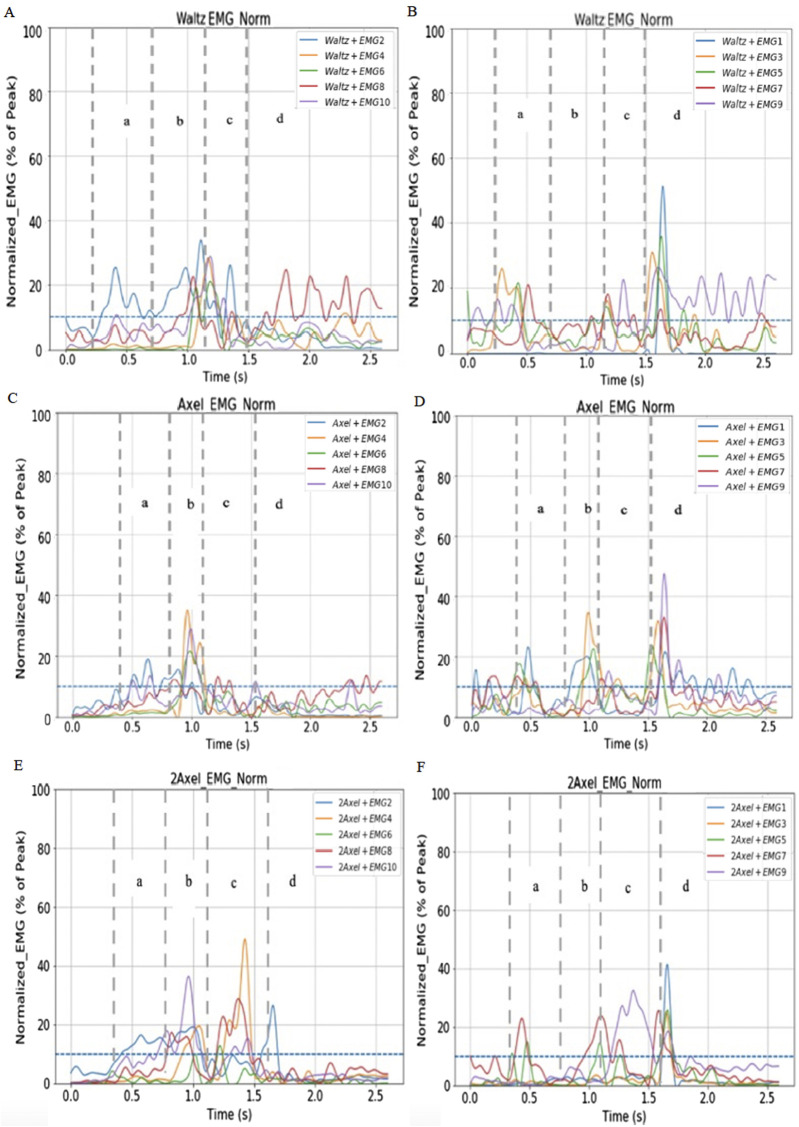
Examples of muscle activity patterns of Axel type jumps from one elite youth skater. **(A,B)** Indicates left and right foot muscles activity during Waltz jump. **(C,D)** Indicates left and right foot muscles activity during Single Axel jump. **(E,F)** Indicates left and right foot muscles activity during Double Axel jump. **(A)** propulsion phase; **(B)** take-off phase; **(C)** flight phase; **(D)** landing phase. EMG 1, 3, 5, 7, 9: Right TA, LG, MG, BF, RF. EMG 2, 4, 6, 8, 10: Left TA, LG, MG, BF, RF.

As shown in Figure 2, take-off leg muscles activated during the waltz jump propulsion phase were following the order of TA and RF. During the take-off phase, the muscle activated following the order of TA, BF, RF, MG and LG. During the landing phase, the muscle activated following the order of LG, RF, MG, TA and BF (See Figures 2A,B). The rest of the athletes had approximately the same muscle activation patterns. Take-off leg muscles activated during the Single Axel jump propulsion phase were following the order of TA and RF. During the take-off phase, the muscle activated following the order of TA, BF, LG, and RF and MG activated at the same time. During the landing phase, the muscle activated following the order of MG, LG, BF, and TA and RF activated at approximately the same time (See Figures 2C,D). Other athletes had approximately the same activation patterns. Take-off leg muscles activated during the Double Axel jump propulsion phase were following the order TA and RF. During the take-off phase, the muscle activated following the order of TA, RF, BF, LG, and MG which the activation was not obvious. During the landing phase, the muscle activated following the order of BF, RF, LG, TA and MG (See Figures 2E,F). Other athletes had approximately the same activation patterns.

### 3.3 Muscle activation levels

#### 3.3.1 Quantifying muscle activation and effort

**FIGURE 3:**
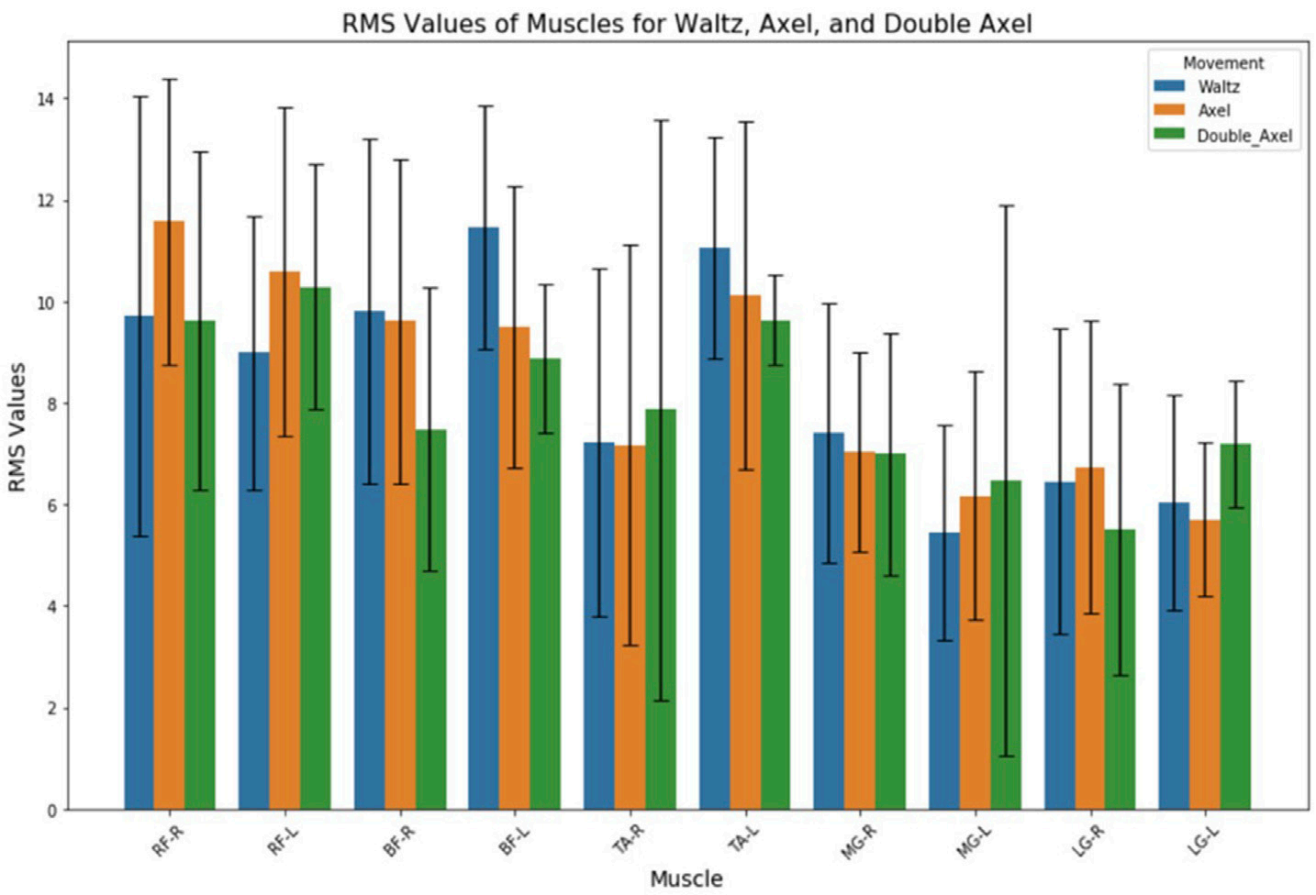
Normalized RMS values of lower limb muscles for Waltz, Single Axel and Double Axel jump RF, rectus femoris; BF, biceps femoris; TA, tibialis anterior; MG, medial gastrocnemius; LG, lateral gastrocnemius.

Normalized RMS values of lower limb muscles for Waltz, Single Axel and Double Axel jumps are shown in Figure 3. Although, there were no significant difference between the normalized RMS values of different muscles during Waltz jump and Single Axel jump, the RMS values of the left MG, left LG and right TA increased as the difficulty of the jump increased. On the other hand, the RMS values of the left BF, left TA and other muscle of the right leg decreased. RMS values of right and left RF were the highest during Single Axel jump and Double Axel jump.

**FIGURE 4:**
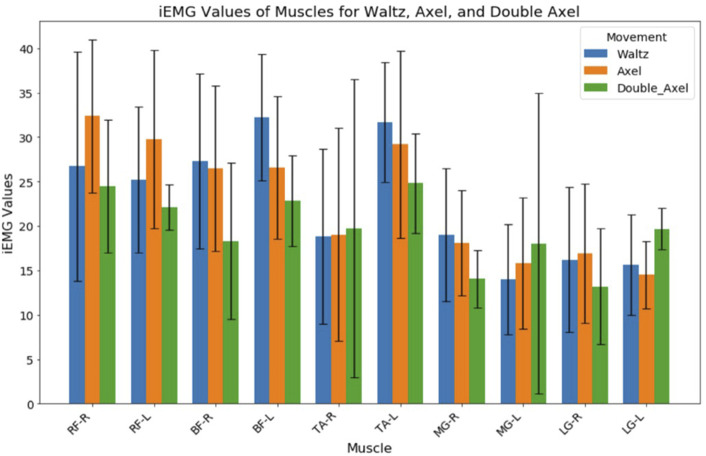
Normalized iEMG Values of Lower Limb Muscles for Waltz, Single Axel and Double Axel Jump RF, rectus femoris; BF, biceps femoris; TA, tibialis anterior; MG, medial gastrocnemius; LG, lateral gastrocnemius.

Normalized iEMG values of the lower limb muscles for Waltz, Single Axel and Double Axel jumps are shown in Figure 4. Although there were no significant differences between the normalized iEMG values of the muscles between the Waltz and Axel jumps, there was an increase in the iEMG value of right foot TA and left MG, and a decrease in both the right and left BF, left TA and right MG as the difficulty of the jumps increased.

#### 3.3.2 Differences in neuromuscular activation across various muscle regions

There were significant differences between different muscle parts RMS values and iEMG values in both Waltz jump and Single Axel jump (p < 0.01). Tukey HSD *post hoc* test results show that, in Waltz jump, biggest differences were found between RMS values of BF-L and MG-L, (differences = 6.03, p = 0.01). The largest and smallest muscle RMS values were at BF-L and MG-L, respectively, during Waltz jump. Moreover, biggest differences were found between iEMG values of BF-L and MG-L (differences = 18.28, p = 0.001). The largest and smallest muscle iEMG values were at BF-L and MG-L, respectively, during Waltz jump. In Single Axel jump, biggest differences were found between RMS values of RF-R and LG-L (differences = 5.88, p = 0.001). The largest and smallest muscle RMS values were at RF-R and LG-L, respectively, during Single Axel jump. Furthermore, biggest differences were found between iEMG values of RF-R and LG-L (differences = 17.88, p = 0.001). The largest and smallest muscle RMS values were at RF-R and LG-L, respectively, during Single Axel jump. With descriptive analysis, largest and smallest muscle RMS values were at RF-L and LG-R (4.78)., respectively, during Double Axel jump. Largest and smallest muscle iEMG values were at TA-L and LG-R (11.62), respectively, during Double Axel jump.

In both Waltz jump and Single Axel jump, the muscles of hamstrings (BF) and quadriceps (RF) indicated to have the highest RMS values and iEMG values, referring to total muscle work and activity duration. BF-L is the most active muscle parts both in terms of muscle activity level and total activated duration during completion of Waltz jump, and RF-R is the most active muscle parts both in terms of muscle activity level and total activated duration during completion of Axel type jumps. However, TA-L seemed to be the muscle most contributed in completion of Double Axel jump.

## 4 Discussion

### 4.1 Spatiotemporal characteristics of Axel type jumps

The waltz jump has the longest take-off time, while the Axel jump, and Double Axel jump have shorter take-off time. The flight time of the Double Axel jump is significantly longer than that of the waltz jump and Axel jump, thus, as the number of rotations increases, jump flight time increases. This result is consistent with the previous studies, where the flight time of Triple Axel is approximately 0.06 s longer than that of Double Axel jump (Hisako et al., 2004) and increasing the maximum jump height was found to be the key factor in mastering the Quadruple Axel jump when comparing the height between Triple Axel and Quadruple Axel (Hirosawa, 2004). Additionally, there were increase in jump height and maximum vertical speed at takeoff, and decrease in jump distance as jump difficulty increases, which indicate the need to optimize energy distribution in the vertical direction during the jump. For some athletes, as jump difficulty increases, the jump distance increase, making the jump appear larger and more aesthetically pleasing, but this may increase the likelihood of falling. Therefore, maximizing jump height while slightly decreasing jump distance could also be the key to land more difficult Axel type jumps. Previous research primarily included only high-level senior athletes, showed that as jump difficulty increases, the rotational speed in the air increases, and the jump distance decreases, which is consistent with the results of this study (King, 2005; King et al., 1994). However, in that study, no differences in jump height and vertical speed at takeoff were found between different jumps, thus more difficult jumps are completed only through an increase in rotational speed in the air, which is inconsistent with this study. Others showed that the average flight time of Triple Axel jumps of Japanese junior female athletes was 0.57 s, with a vertical speed at takeoff of 2.63 m/s and a jump height of 0.36 m (Hisako et al., 2004). Which is 0.06 s longer than the average flight time of 0.51 s of the Double Axel jump in this study, and the vertical speed at takeoff is 0.1 m/s slower (2.73 m/s), with the same jump height. This indicates that the Triple Axel jump might be completed by generating more efficient angular momentum, tightening the arms and legs as much as possible, reducing rotational resistance, lowering the moment of inertia, and increasing angular velocity. The differences in results may be influenced by the subject group. It can be inferred that the technical movements of high-level athletes are more refined, and their ability to control inertial forces and increase angular velocity is higher, meaning that high-level athletes can stop the arm and leg movements after the propulsion phase and then quickly bring the arms and legs closer to the rotation axis through the take-off and during the jump.

In addition, previous study has reported Double Axel jumps with greater jump distance have more likelihood of receiving better Grade of Execution (GOE) points by the judges (Hirosawa et al., 2022). Combining the result of this study, figure skaters are recommended to increase their jump height and take-off vertical velocity first to acquire difficult jumps, and later improve jump distance and the overall quality of jumps by increasing the horizontal speed before the execution of jumps.

### 4.2 Lower limb joint angle characteristics of Axel type jumps

In this study, hip angle at propulsion phase during Single Axel jump showed significantly larger flexion angle than during Waltz jump. Overall, there were increases in hip ROM during both propulsion and take-off as the jump difficulty increases, indicating that athletes are required to fully utilize the flexion of the lower-limb joints during the propulsion phase and maximize hip joint extension at takeoff to complete difficult jump, which supports our first hypothesis. Highest knee ROM at takeoff during Double Axel jump were found; however, no clear patterns of increasing knee ROM were observed between Waltz jump and Single Axel jump. Therefore, our second hypothesis is not supported. Larger joint movements create a spring-like effect, allowing the athlete to jump higher. Thus, this study shows that jumps with more rotations are completed by reducing the hip, knee and ankle joint angles during the propulsion phase, which is consistent with previous studies that showed an increase in jump height is achieved by reducing the ankle joint angle in the propulsion phase (Iwanska et al., 2018). Higher knee and hip joint flexion range during more difficult jumps suggests that there can be increases in knee and hip injuries; therefore, strengthening programs targeting lower limb are extremity important before attempting difficult jumps on ice.

More difficult jumps require athletes to accumulate energy and perform the takeoff action in a shorter period while generating enough flight time to complete more rotations in the air. To successfully perform more difficult Axel jumps, athletes need to enhance their lower body vertical explosive power, ability to increase jump height, extend flight time, and to convert linear motion into rotational motion. The use of the lower-limb joints (hip, knee, and ankle) during propulsion phase of the jump is crucial. The knee joint angles at takeoff for Double Axel jumps in both on ice and off-ice training conditions are very similar (Mazurkiewicz and Iwanska, 2015), suggesting that youth athletes can optimize the takeoff movement of the Axel jump through off-ice jump training. For junior athletes, premature pre-rotation is a common issue that can negatively affect jump height, the number of rotations, and landing stability (Liu, 2014). The proper technical sequence of the Axel jump includes down swing, upswing, ice push-off, and rotation. Therefore, the Axel jump must be performed with a proper rhythm to ensure technical stability, jump success rate and lower injury rates.

### 4.3 Muscle activity patterns and activity levels during Axel type jumps

As the jump difficulty increased, the muscle activation pattern shifted from a wider distribution to a more efficient pattern. First, activation pattern of the major muscles in the propulsion phase remained consistent across three jumps, with the take-off leg TA and RF dominating. The take-off leg RF and TA remain as the primary muscles in the propulsion and take-off phases, and the intensities of these muscle activations remain high in all three jumps. RF of the take-off leg was activated to control the rate of knee flexion, rapid joint extension, and joint protection during the propulsion and take-off phases. TA was activated to adjust foot contact with ice, shifting weight distribution and slowly control ankle dorsiflexion. This suggests that these muscles play an important role in energy accumulation and upward momentum generation during the jump. Secondly, the activation pattern of TA, BF, and then calf muscles during the take-off phase was consistent across jumps. Activation of BF and MG increased during the take-off phase compared with the propulsion phase. BF assists in the extension of the hip joints and stabilize the lower body in take-off phase. MG aids in plantarflexing the ankle to provide the final upward thrust of the jump. The activation pattern of muscles in the landing phase of different jumps showed some differences. Landing leg BF and RF tend to be activated earlier with increasing difficulty, indicating that at the beginning of landing of more difficult jumps requires better knee stability and hip mobility, to stabilize the high impact of the landing from higher jump height in difficult jumps. During the landing phase, there is increased activation of all muscles of the landing leg. Muscle activation patterns indicate that the landing leg muscles need to be trained evenly to stabilize the landing for jumping. Although not statistically different, muscle activation of the RF appears to increase during the takeoff phase of jumps with more rotations. In addition, muscle activation of MG muscle appears to increase during the overall jump, which is consistent with previous research on Axel jumps in roller skating (Pantoja et al., 2014). The results for the other muscle areas differed from other research and may be due to differences in subject populations. These results partially support our third hypothesis as some muscle parts activation levels increase during as jump rotation increases; however, the findings are not conclusive enough to fully validate the hypothesis.

More difficult jumps required higher levels of muscle coordination. Although the primary muscle groups during the propulsion, take-off and landing phases of the jumps were generally consistent, there were some differences in the pattern of secondary muscle involvement between the Waltz and Axel jumps, and Double Axel jump. Smaller activation level of the LG muscle during the take-off phase of the Double Axel jump suggests that the jumps require a more efficient activation pattern, which would require the athletes to be able to activate different parts of the muscle separately rather than synchronizing them. Only the needed muscles are activated, and the unwanted muscles are not as activated. In addition, the landing leg RF and TA are always involved as primary muscles in the landing phase, but their activation levels are significantly higher in the Waltz and Axel jumps and slightly lower in the Double Axel jump, indicating a greater reliance on the precise control of these muscles to absorb impact and maintain stability. This high demand for ice landing stability and related muscle activation patterns alternation demonstrates the technical complexity of difficult jumps. Particularly in youth athletes, improper landing mechanics and neuromuscular fatigue can lead to overuse injuries. Thus, understanding these muscle activation patterns and their relationship to landing mechanics are essential for developing targeted injury prevention strategies and optimizing performance.

The RMS and iEMG values of the left MG, left LG and right TA increased as the jump difficulty increased, whereas the values of the left BF and the right gastrocnemius muscles decreased. The differences in the activation of these muscles may be mainly in the take-off and flight phase, as the Double Axel jump has the longest flight time and more rotations. Double Axel jump requires a high level of coordination and postural stability of the muscles of the lower limbs during the flight phase, and these muscles play an important role in the postural and rotational control.

There was a correlation found between countermovement jump height with angle of the pelvis during propulsion and angle of pelvis and knee during take-off phase (Endy et al., 2017). Moreover, there is also a relationship between figure skating jump height and muscle strength especially for knee extension (Podolsky et al., 1990), and those skaters who can perform Double Axel jumps were also found to perform greater knee extension and plantar flexion (Comuk and Erden, 2012). Hamstrings are responsible for knee flexion and hip extension (Park and Lim, 2023; Avers and Brown, 2018), and quadriceps for knee extension (Bordoni and Varacallo, 2023). These findings align with this study results, since highest muscle activities were found at hamstrings and quadriceps in all jumps. Also, increase in medial gastrocnemius activation were found in more difficult jumps, all of which reflects that these muscles are required to generate the primary force, power, explosive energy, and stabilized lower body through greater knee, hip and ankle joint controls, for optimal jumping performance. These muscles work synergistically to produce stable, rapid, coordinated movements during jump propulsion and jump take-off for maximizing jump height and maximal take-off vertical velocity while maintaining control and efficiency.

Muscle recruitment pattern also highlights the importance of strength training and neuromuscular coordination in enhancing jump performance. From these results, it is suggested that figure skaters need to improve large muscle group strength and lower limb coordination for acquiring difficult jumps. Strengthening the rectus femoris and biceps femoris are necessary to enhance hip and knee control for jumps. Training methods include lunge jumps, deep squats, glute bridges and single leg pulls can be useful to optimize the power output of the large muscle groups including rectus femoris and biceps femoris. Secondly, athletes need to improve the efficiency of the power chain of the lower limbs, hip-knee-ankle joints, optimize joint coordination and improve jumping techniques. Training methods include box jumps, deep jumps, and drop jumps, all focusing on coordinated hip-knee-ankle power output, improving cushioning and joint coordination when landing. Balance board exercises, single leg calve raises, and other foot stability exercises can be used to enhance ankle stability, increase calf muscle function, balance and endurance, and improve muscle activation patterns of the tibialis anterior and other muscle coordination during the propulsion phase while avoiding over-activation due to fatigue. These training methods can help youth athletes to not only jump better but also reduce injury risks.

## 5 Limitation

This study provides a preliminary examination of lower-limb biomechanical characteristics of Axel type jumps in youth figure skaters; however, there are several limitations. Firstly, the overall sample size is relatively small, which may have an impact on the data statistics and limit the generalizability of the results of the present work. Due to the lack of high-level junior figure skaters with stable jumping skills, the sample size of Double Axel jump was especially small. Due to the limited sample size of Double Axel (n = 3), all findings related to the Double Axel are purely descriptive and should be interpreted as exploratory. Secondly, there are many other factors affecting the performance of skaters, such as gender, age, training background, which in this study did not consider. Gender-differences in jumping performance are commonly observed in youth athletes (Magnúsdóttir et al., 2011; Atkinson et al., 2024). In pair figure skaters, gender differences in vertical jump characteristics during repetitive jump test were also reported (Sands et al., 2012). There are differences in the typical injury profiles between male and female skaters (Dubravcic-Simunjak et al., 2003). These findings suggest that there may be gender differences in biomechanical characteristics of Axel type jumps. Future study can include more samples, with same sample size of both male and female to perform gender-based subgroup analysis and analyze gender-based biomechanical differences in figure skating jump performance. In addition, body mass and height, which may influence both EMG amplitude and joint kinematics. Future studies with larger sample sizes should consider incorporating covariate adjustments, such as body mass and height, and including such adjustments through ANCOVA or regression-based methods to improve the precision of biomechanical comparisons in youth athletes. More higher-level skaters who acquire Double Axel jump and even more difficult jumps such as Triple Axel can be included for more in-depth jump analysis. Also, a detailed analysis of the flight phase to characterize associated biomechanics of jump flight position and posture can also be conducted in the future.

Despite these constraints, this study explored the kinematic characteristics and muscular activation characteristics of figure skating Axel type jumps with different number of rotations in youth skaters. Uniquely identified which muscles are more involved in figure skating jumps, which could be helpful for designing specific training programs for strengthening these muscles, which may provide benefits to youth athletes.

## 6 Conclusion

This study analyzed lower-limb kinematics and neuromuscular activity characteristics of Axel type jumps in youth figure skaters and compared characteristics between jumps with different difficulty including Waltz jump, Single Axel jump and Double Axel Jump. The results indicate that skaters need to apply greater lower limb flexion, and while maintaining greater activities in hamstrings and quadriceps before take-off, using more of tibialis anterior, lateral gastrocnemius, and medial gastrocnemius throughout jumps can be important to jump higher, faster take-off vertical velocity, and to obtain enough time in air to complete jumps with additional rotations. In conclusion, figure skaters are required to enhance lower limb flexibility, stability, proper joint and muscular coordination, to acquire more difficult jumps that are with more rotations. This study uniquely identified the similarities and differences in the lower-limb biomechanical characteristics across different types of Axel jumps by youth figure skaters. It also explored the technical and neuromuscular strategies youth skaters employ to master more complex Axel jumps. These insights can be valuable for both youth figure skaters and coaches in developing training programs and injury prevention strategies for figure skating jumps.

## Data Availability

The raw data supporting the conclusions of this article will be made available by the authors, without undue reservation.

## References

[B1] Atkinson M. A. James J. J. Quinn M. E. Senefeld J. W. Hunter S. K. (2024). Sex differences in track and field elite youth. Med. Sci. sports Exerc. 56 (8), 1390–1397. 10.1249/MSS.0000000000003423 38595163

[B2] Avers D. Brown M. (2018). Daniels and worthingham’s muscle testing E-Book: techniques of manual examination and performance testing. St. Louis, Missouri: Saunders (an imprint of Elsevier) Elsevier Health Sciences.

[B3] Baker J. (2003). Early specialization in youth sport: a requirement for adult expertise? High. Abil. Stud. 14 (1), 85–94. 10.1080/13598130304091

[B4] Bordoni B. Varacallo M. A. (2023). “Anatomy, bony pelvis and lower limb: Thigh quadriceps muscle,” in StatPearls (Treasure Island (FL): StatPearls Publishing). Available online at: https://pubmed.ncbi.nlm.nih.gov/30020706/. 30020706

[B5] Bruening D. A. Reynolds R. E. Adair C. W. Zapalo P. Ridge S. T. (2018). A sport-specific wearable jump monitor for figure skating. PLoS One 13 (11), e0206162. 10.1371/journal.pone.0206162 30462651 PMC6248918

[B6] Cattle A. Mosher A. Mazhar A. Baker J. (2023). Early specialization and talent development in figure skating: elite coaches’ perspectives. Curr. Issues Sport Sci. 8 (1), 013. 10.36950/2023.1ciss013

[B7] Cohen E. J. Bravi R. Minciacchi D. (2017). 3D reconstruction of human movement in a single projection by dynamic marker scaling. PLoS ONE 12 (10), e0186443. 10.1371/journal.pone.0186443 29045439 PMC5646814

[B8] Comuk N. Erden Z. (2012). The effect of muscular strength and endurance on technical skill in professional figure skaters. Isokinet. Exerc. Sci. 20 (2), 85–90. 10.3233/IES-2012-0445

[B9] Dubravcic-Simunjak S. Pecina M. Kuipers H. Moran J. Haspl M. (2003). The incidence of injuries in elite junior figure skaters. Am. J. Sports Med. 31 (4), 511–517. 10.1177/03635465030310040601 12860537

[B10] Endy U. Damayanti T. Pawana I. Dwikora U. (2017). Relationship for knee angle, hip angle and peak ground reaction force with vertical jump performance at volleyball athlete in surabaya. Int. Meet. Regen. Med., 321–329. 10.5220/0007321003210329

[B11] Hermens H. J. Freriks B. Disselhorst-Klug C. Rau G. (2000). Development of recommendations for SEMG sensors and sensor placement procedures. J. Electromyogr. Kinesiol. 10, 361–374. 10.1016/S1050-6411(00)00027-4 11018445

[B12] Hines J. R. (2006). Figure skating: a history. Urbana, Illinois: University of Illinois Press.

[B13] Hirosawa S. (2004). Kinematic considerations for achieving the quadruple axel jump: comparison with triple axel jumps among world-class figure skaters using tracking data. Sports Biomech. 10 (1-12), 1–12. 10.1080/14763141.2025.2464787 39950336

[B14] Hirosawa S. Watanabe M. Aoki Y. (2022). Determinant analysis and developing evaluation indicators of grade of execution score of double axel jump in figure skating. J. Sports Sci. 40 (4), 470–481. 10.1080/02640414.2021.1997407 34781855

[B15] Hisako I. Yasuo I. Shinji S. (2004). Biomechanics of jump in figure skating – kinematic analysis of triple axel jump motion of women’s figure skaters. Nagoya J. Health, Phys. Fit. Sport. 27, 1.

[B16] International Skating Union (2024). SINGLE and PAIR SKATING levels of difficulty and guidelines for marking grade of execution and program components. Available online at: https://www.isu.org/isu-communications/?tab=ISU%20Communications.

[B17] Iwanska D. Mazurkiewicz A. Urbanik C. (2018). Biomechanics of the axel paulsen figure skating jump. Pol. J. Sport Tour. 25, 3–9. 10.2478/pjst-2018-0007

[B18] Jederström M. Agnafors S. Ekegren C. Fagher K. Gauffin H. Korhonen L. (2021). Determinants of sports injury in young female Swedish competitive figure skaters. Front. Sports Act. Living 3, 686019. 10.3389/fspor.2021.686019 34222861 PMC8253259

[B19] King D. L. (2005). Performing triple and quadruple figure skating jumps: implications for training. Can. J. Appl. Physiol. 30 (6), 743–753. 10.1139/h05-153 16485524

[B20] King D. L. Arnold A. S Smith A. (1994). A kinematic comparison of single, double, and triple axels. J. Appl. Biomech. 10, 51–60. 10.1123/jab.10.1.51

[B21] Kos A. Umek A. (2019). Wearable sensor devices for prevention and rehabilitation in healthcare: swimming exercise with real-time therapist feedback. IEEE Internet Things J. 6 (2), 1331–1341. 10.1109/JIOT.2018.2850664

[B22] Liu Y. (2014). Some common technical diagnosis and correction of aksay jump in figure skating. China Winter Sports 36 (4).

[B23] Lockwood K. L. Gervais P. J. Mccreary D. R. (2006). Landing for success: a biomechanical and perceptual analysis of on-ice jumps in figure skating. Sports Biomech. 5 (2), 231–241. 10.1080/14763140608522876 16939155

[B24] Maddox E. U. Bennett H. J. Weinhandl J. T. (2022). Evidence for the use of dynamic maximum normalization method of muscle activation during weighted back squats. J. Biomech. 135, 111029. 10.1016/j.jbiomech.2022.111029 35272129

[B25] Madsen A. Alfonso K. Vincent H. K. (2024). Figure skating musculoskeletal injury: evidence across disciplines, mechanisms, and future directions. Curr. Sports Med. Rep. 23 (10), 332–339. 10.1249/JSR.0000000000001198 39514724

[B26] Magnúsdóttir H. Sveinsson T. Árnason A. (2011). Gender difference in jumping and landing among 15–18-year old icelandic national youth soccer players. Br. J. Sports Med. 45, 361. 10.1136/bjsm.2011.084038.145

[B27] Mazurkiewicz A. Iwanska D. (2015). Biomechanics of figure skating jump double axel performed in on ice and off ice conditions. Aktual. Probl. Biomech. 9, 83–88. 10.2478/pjst-2021-0007

[B28] Pantoja P. D. Mello A. Liedtke G. V. Kanitz A. C. Cadore E. L. Pinto S. S. (2014). Neuromuscular responses of elite skaters during different roller figure skating jumps. J. Hum. Kinet. 41 (1), 23–32. 10.2478/hukin-2014-0029 25114728 PMC4120457

[B29] Park S. Lim W. (2023). Comparison of muscle activity of hamstrings as knee flexors and hip extensors and effect of tibial and hip rotation on the contribution of hamstrings. J. Bodyw. Mov. Ther. 34, 1–5. 10.1016/j.jbmt.2023.04.029 37301549

[B30] Podolsky A. Kaufman K. R. Cahalan T. D. Aleshinsky S. Y. Chao E. Y. (1990). The relationship of strength and jump height in figure skaters. AJSM 18 (4), 400–405. 10.1177/036354659001800412 2403190

[B31] Pueo B. (2016). High speed cameras for motion analysis in sports science. J. Hum. Sport Exerc. 11, 53–73. 10.14198/jhse.2016.111.05

[B32] Rauer T. Pape H. C. Knobe M. Pohlemann T. Ganse B. (2022). Figure skating: increasing numbers of revolutions in jumps at the European and world championships. PLoS One 17 (11), e0265343. 10.1371/journal.pone.0265343 36449462 PMC9710745

[B33] Sands W. A. Kimmel W. L. McNeal J. R. Murray S. R. Stone M. H. (2012). A comparison of pairs figure skaters in repeated jumps. J. sports Sci. and Med. 11 (1), 102–108. Available online at: https://pmc.ncbi.nlm.nih.gov/articles/PMC3737852/. 24149126 PMC3737852

[B34] Taborri J. Keogh J. Kos A. Santuz A. Umek A. Urbanczyk C. (2020). Sport biomechanics applications using inertial, force, and EMG sensors: a literature overview. Appl. Bionics Biomech. 2020, 1–18. 10.1155/2020/2041549 PMC7330631 32676126

[B35] Taylor C. L. Psycharakis S. G. (2009). A pilot study on electromyographic analysis of single and double revolution jumps in figure skating. J. Exerc. Sci. Physiother. 5 (1), 14–19. Available online at: http://www.efha.in/wp-content/uploads/2015/01/JESP-5-14.pdf.

